# Optimal alpha reduces error rates in gene expression studies: a meta-analysis approach

**DOI:** 10.1186/s12859-017-1728-3

**Published:** 2017-06-21

**Authors:** J. F. Mudge, C. J. Martyniuk, J. E. Houlahan

**Affiliations:** 1 Department of Biology, Canadian Rivers Institute, University of New Brunswick, Saint John, NB E2L 4L5 Canada; 2 Center for Environmental and Human Toxicology & Department of Physiological Sciences, UF Genetics Institute, University of Florida, Gainesville, Florida, 32611 USA

**Keywords:** Microarrays, RNA-seq, Type I and II error rates, High throughput analysis, Multiple comparisons, Post-hoc corrections, Optimal α

## Abstract

**Background:**

Transcriptomic approaches (microarray and RNA-seq) have been a tremendous advance for molecular science in all disciplines, but they have made interpretation of hypothesis testing more difficult because of the large number of comparisons that are done within an experiment. The result has been a proliferation of techniques aimed at solving the multiple comparisons problem, techniques that have focused primarily on minimizing Type I error with little or no concern about concomitant increases in Type II errors. We have previously proposed a novel approach for setting statistical thresholds with applications for high throughput omics-data, optimal α, which minimizes the probability of making either error (i.e. Type I or II) and eliminates the need for post-hoc adjustments.

**Results:**

A meta-analysis of 242 microarray studies extracted from the peer-reviewed literature found that current practices for setting statistical thresholds led to very high Type II error rates. Further, we demonstrate that applying the optimal α approach results in error rates as low or lower than error rates obtained when using (i) no post-hoc adjustment, (ii) a Bonferroni adjustment and (iii) a false discovery rate (FDR) adjustment which is widely used in transcriptome studies.

**Conclusions:**

We conclude that optimal α can reduce error rates associated with transcripts in both microarray and RNA-seq experiments, but point out that improved statistical techniques alone cannot solve the problems associated with high throughput datasets – these approaches need to be coupled with improved experimental design that considers larger sample sizes and/or greater study replication.

**Electronic supplementary material:**

The online version of this article (doi:10.1186/s12859-017-1728-3) contains supplementary material, which is available to authorized users.

## Background

Microarrays and next generation sequencing (NGS) have been described as technological advances that provide global insight into cellular function and tissue responses at the level of the transcriptome. Microarray and NGS are used in experiments in which researchers are testing thousands of single-gene hypotheses simultaneously. In particular, microarrays and NGS are often used to test for differences in gene expression across two or more biological treatments. These high-throughput methods commonly use *p*-values to distinguish between differences that are too large to be due to sampling error and those that are small enough to be assumed to be due to sampling error. There is little doubt that microarrays/NGS have made a large contribution to our understanding of how cells respond under a variety of contexts, for example in environmental, developmental, and the medical sciences [1–3].

High throughput methods have, however, made interpretation of hypothesis testing more difficult because of the large number of comparisons that are done in each experiment [4]. That is, researchers will examine the effects of one or more treatment on the abundance of 1000s of transcripts. For each gene, there will be replication and a null hypothesis test of whether there is a statistically significant difference in relative expression levels among treatments. In most cases, the statistical threshold for rejecting the null hypothesis (i.e. α) is α = 0.05 although it may occasionally be set at a lower value such as 0.01 or 0.001. Thus, for any individual comparison, the probability of rejecting the null hypothesis when it is true is 5% (if the threshold is set at 0.05). When multiple tests are conducted on 1000’s of transcripts, this creates the potential for hundreds of false positives (i.e. Type I error) at the experiment-wide scale, with the expected number of false positives depending on both the number of tests conducted (known) and the number of those tests where the treatment has no effect on gene expression (unknown). Researchers identified this problem early on and have used a variety of post-hoc approaches to controlling for false positives [5–9].

Approaches for adjusting *p*-values and reducing false ‘positives’ when testing for changes in gene expression, such as Bonferroni or Benjamini-Hochberg procedures, are designed to control experiment-wide error probabilities when many comparisons are being made. Typically they reduce the α for each test to a value much smaller than the default value of 0.05, so that the experiment-wide error is not as inflated due to the large number of comparisons being made. They all share the characteristic that they only explicitly address probabilities of Type I errors [4]. This has the effect of increasing the probability of false negatives (i.e. Type II errors) to varying degrees. This focus on Type I errors implies that it is much worse to conclude that gene expression is affected by a treatment when it is not than to conclude that expression is not affected by a treatment when, in reality, it is. Although there has been some focus on methods designed to balance Type I and Type II error rates [10], researchers rarely discuss the Type II implications of controlling Type I errors, and we believe this suggests that most researchers simply are not considering the effect of post-hoc adjustments on Type II error rates. Krzywinski and Altman [4] note the problem and offer practical advice, “we recommend always performing a quick visual check of the distribution of P values from your experiment before applying any of these methods”. Our position is that this does not go far enough; we assert that post-hoc corrections to control Type I errors don’t make sense unless (1) the researcher knows their Type II error probability (i.e. power) and (2) has explicitly identified the relative costs of Type I and II errors. We have recently developed a solution, optimal α, that balances α (the acceptable threshold for Type I errors – usually 0.05) and β (the acceptable threshold for Type II errors – often 0.20 but the standard practice is more variable than for α), minimizing the combined error rates and eliminating the need for any post-hoc adjustment [11–13]. In the context of transcriptomics, this reduces the overall error rate in identifying differentially expressed genes by finding the best trade-off between minimizing false detections of differential expression and minimizing nondetection of true differential expression.

While we have demonstrated this approach in the context of detecting environmental impacts of pulp and paper mills [13], it is of particular value in fields such as transcriptomics where many tests are conducted simultaneously. While microarrays and RNA-seq have been tremendous technological advances for transcriptomics, when coupled with low sample sizes, it magnifies multiple comparisons problems. The objectives of this paper were to apply optimal α to a set of published microarray data to demonstrate that using the optimal α approach reduces the probabilities of making errors and eliminates the need for any post-hoc adjustments. In addition, we discuss modifications to the experimental design of microarray data that directly address the problem of multiple comparisons.

## Methods

### Data collection

We collected data on microarray experiments conducted in teleost fishes spanning a period of 10 years (see Additional file 1: Data S1). Environmental toxicology is the research focus of one of the authors, however we point out here that this approach is not confined to aquatic toxicology and is applicable across disciplines. The search for microarray fish studies was conducted from January 2011–August 2011 using the search engines *Web of Science, Science Direct, PubMed (National Center for Biotechnology Information),* and *Google Scholar*. Keywords and combinations of key words used in the search engine included “microarray”, “gene expression”, “DNA chip”, “transcriptomics”, “arrays”, “fish”, “teleost”, and “aquatic”*.* In addition, references from papers were reviewed for information on manuscripts not identified by the search engines. This intensive search resulted in representation of studies encompassing a wide range of teleost fishes and scientific disciplines (e.g. physiology, toxicology, endocrinology, and immunology). There were a total of 242 studies surveyed for information (Additional file 1: Data S1).

The extracted data from microarray experiments included fish species, family, sex, analyzed entity (e.g. cell, tissue), experimental treatment, concentration (if applicable), duration, exposure type, microarray platform, type of normalization, number of biological replicates, endpoints assessed, number of differentially expressed genes (DEGs) identified by the researchers, total gene probes on the array, average fold change of DEGs, and the method of post hoc analysis. Approximately 50% of these studies applied an FDR threshold as the method of choice for detecting differentially expressed genes. All microarray data were normalized by the authors of the original studies using the method of their choice (there are different methods but they differ only slightly).

### Calculating optimal Α

For each study, we calculated optimal α levels [11] that minimized the combination of Type I and Type II error probabilities, and compared the Type I and II error probabilities resulting from this approach to those associated with using α = 0.05. Data are summarized on a per-paper, not on a per test basis.

The calculation of an optimal α level requires information concerning the test type, the number of replicates, the critical effect size, the relative costs of Type I vs. Type II errors, and the relative prior probabilities of null vs. alternate hypotheses. Optimal α calculations are based on minimizing the combined probability or cost of Type I and II errors by examining the mean probability of making an error over the entire range of possible α levels (i.e. from 0 to 1). This is a 5 step process. Step 1 – Choose an α level between 0 and 1. Step 2 – Calculate β for the chosen α, sample size, critical effect size and variability of the data (this can be achieved using a standard calculation of statistical power for the statistical test being used, beta is 1 – statistical power), Step 3 – Calculate the mean of α and β, Step 4 – Choose a new α slightly smaller than the previous α and compare the mean error probability with the previous iteration. If it is larger choose a new α slightly larger than the previous α. If it is smaller choose a new α slightly smaller than the current α, Step 5 – Keep repeating until the improvement in mean error probability fails to exceed the chosen threshold – at this stopping point you have identified optimal α. Several assumptions or constraints were made to enable consistent optimal α analysis of studies with a wide degree of technical and statistical methodologies:

### Assumptions

(1) We used the number of biological replicates in each group as the level of replication in each study. Microarrays were sometimes repeated on the same biological replicates but this was not treated as true replication, regardless of whether it was treated as replication within the study. Similarly, spot replicates of each gene on a microarray were not treated as replication, regardless of whether it was treated as replication within the study There were 39 studies which had levels of biological replication of *n* = 1, or *n* = 2. These studies were omitted from further statistical analysis, leaving 203 studies with biological replication of *n* ≥ 3.

(2) Two hundred and three of the 242 studies identified were suitable for analysis and to ensure that the optimal α value in all 203 studies were calculated on the same test and are comparable, we analyzed each study as an independent, two-tailed, two-sample t-test even though some studies used confidence interval, randomization or Bayesian analyses instead of t-tests. ANOVA was also occasionally used instead of t-tests but even in cases where ANOVA was used, it was the post-hoc pairwise comparisons between each of the experimental groups and a control group that were the main focus. These post-hoc pairwise comparisons are typically t-tests with some form of multiple comparison adjustment. One-tailed or paired t-tests were sometimes used instead of two-tailed independent tests. Although these tests do increase power to detect effects, they do so by placing restrictions on the research question being asked.

(3) Critical effect size, in the context of t-tests, is the difference in the endpoint (in this case, gene expression) between treatment and control samples that you want to detect. In traditional null hypothesis testing, β is ignored and critical effect sizes are not explicitly considered. We calculated optimal α levels at three potential critical effect sizes, defined in terms relative to the standard deviation of each gene (1 SD, 2 SD and 4 SD). Fold-changes or percent changes from the control group were occasionally used as critical effect sizes, but we avoided these effect sizes to maintain consistency across optimal α calculations and because we believe the difference in expression relative to the variability in the gene is more important than the size of effect relative to the control mean of the gene. A two-fold change in a gene may be well within the natural variability in expression of one gene and far outside the natural variability in expression of another gene. However, there may be contexts where fold-changes are more appropriate than standard deviations and optimal alpha can accommodate this by setting separate optimal alphas for each gene. Separate thresholds would be required because detecting a 2-fold change in a highly variable gene would result in a larger optimal alpha than detecting a 2-fold change a gene with little variability.

(4) We assumed the relative costs of Type I and Type II errors to be equal, representing a situation where researchers simply want to avoid errors, regardless of type. However, optimal alpha can accommodate any estimates of the relative costs of Type I and II errors (See code in Additional file 2: Appendix S1). So, where there is clear evidence of different relative Type I and II error costs they should be integrated into optimal alpha estimates. Multiple comparison adjustments that reduce Type I error rates without (1) estimating Type II error probability and (2) the relative costs of Type I and II errors are ill-advised.

(5) We assumed that the prior probabilities for the meta-analysis and the required within and among-study replication to be - H_A_ prior probability = 0.50 and H_o_ prior probability = 0.50. For the simulations comnparing optimal alpha error rates relative to traditional multiple comparisons approaches we used three prior probability scenarios - Scenario 1: H_A_ prior probability = 0.50 and H_o_ prior probability = 0.50, Scenario 2: H_A_ prior probability = 0.25 and H_o_ prior probability = 0.75, and Scenario 3: H_A_ prior probability = 0.10 and H_o_ prior probability = 0.90. There has been relatively little empirical work done describing the proportion of genes that are affected by treatments in microarray studies but [14] examined the effects of mutations in different subunits of the transcriptional machinery on the percent of genes that showed differential expression and concluded that the percent of genes ranged from 3 to 100% with a mean of 47.5%. Another estimate by Pounds and Morris [15] suggested that slightly more than half the genes in a study examining two strains of mice showed differential gene expression. In addition, accurate estimation of global gene expression has been complicated by inappropriate assumptions about gene expression data [16, 17] and further research in this area is critical. There is no way of being certain of how many true positives and true negatives there are in each study but in the absence of any prior knowledge the rational assumption is that the probabilities are equal (Laplace’s principle of indifference). However, in the context of gene expression a differential expression prior probability of 0.50 is at the high end and so we also examined H_A_ prior probabilities of 0.25 and 0.10. Prior probabilities other than equal can be accommodated by optimal α and using other prior probabilities would result in quantitative differences in the results. However, the general conclusion that optimal alpha error rates will always be as low or lower than traditional approaches does not depend on the assumed prior probabilities.

### Analyses

*Minimum average of α and β for each of the 203 studies at 3 different critical effect sizes (1, 2, and 4 SD’s):* We calculated the average of α and β using optimal α and the traditional approach of setting α = 0.05. To do this we calculated optimal α for each of the 203 studies as described above, extracted the β associated with optimal α for each of the 203 studies, and calculated the average of α and β. Similarly, we extracted the β associated with α = 0.05 for each of the 203 studies when and then calculated the average of α and β. We could then compare the average of α and β for optimal α and α = 0.05.

#### Effect of post-hoc corrections on error rates (see Additional file 3: Data S2)

We simulated 15,000 tests of the effect of a treatment for each of 3 prior probability scenarios and 3 effect size scenarios. The prior probability scenarios were Scenario 1: H_A_ prior probability = 0.50 and H_o_ prior probability = 0.50, Scenario 2: H_A_ prior probability = 0.25 and H_o_ prior probability = 0.75, and Scenario 3: H_A_ prior probability = 0.10 and H_o_ prior probability = 0.90. The effect size scenarios were Scenario 1: 1 SD, Scenario 2: 2 SD, and Scenario 3: 4 SD. All comparisons were made using two-tailed, two-sample t-tests. Based upon experience and the literature, gene expression studies vary widely in the proportion of genes that are differ entially expressed and usually show a small effect (1 SD). We only select larger values (2 and 4 SD) above to illustrate the application of the optimal α compared to other post-hoc tests. All differences between treatment and control were chosen from normal distributions that reflected the ‘true’ differences (i.e. 0, 1, 2 or 4 SD’s). We calculated error rates using optimal α, α = 0.05, α = 0.05 with a Bonferroni correction and α = 0.05 with a Benjamini-Hochberg False Discovery Rate correction. We then compared the total number of errors across all 15,000 tests for the four different approaches. For example, using Scenario 1 for both effect size and prior probability we compared the 4 approaches under the assumption that half the genes were affected by the treatment and the size of the effect for those 7500 genes was 1 SD. By contrast, using Scenario 3 for both prior probability and effect size we assumed that 1500 of the genes were differentially expressed and the size of the effect was 4 SD.

#### Minimum number of within-study replicates needed to meet desired error rate

The same iterative process that can be used to calculate minimum average error rate for a specific sample size can be used to calculate minimum sample size for a specific average error rate. Here we identified a range of minimum acceptable average error rates from 0.00001 to 0.125 (reflecting the common practice of α = 0.05 and β = 0.2) and calculated the minimum sample size required to achieve the desired error rates for 3 different effect sizes (i.e. 1, 2, and 4 SD’s).

#### Minimum among-study replication needed to meet desired error rate

An alternative to large within-study replication is to synthesize similar studies that have been replicated several times. Here we simply identified how often a study would have to be repeated at a specific optimal α to achieve a desired error rate. For example, to detect a 1 SD difference between treatment and control using a 2-sample 2-tailed t-test with a sample size of 4 the optimal α is 0.29. If optimal α is 0.29 but the desired error rate is 0.00001 we solve for x in 0.00001 = 0.29^x and conclude that 10 studies showing a significant difference between treatment and control expression would be necessary to meet our desired threshold. Similarly, β at this optimal α is 0.38 and we would need 12 studies showing no significant difference between treatment and control to meet our desired error rate.

## Results

### Meta-analysis

**Fig. 1:**
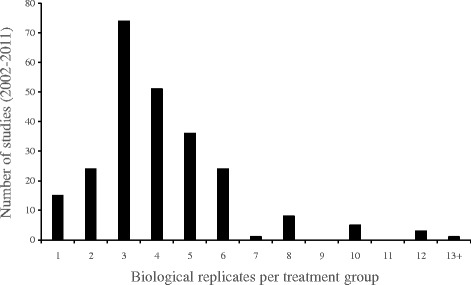
Distribution of the number of biological replicates per treatment group over 203 fish microarray papers published between 2002 and 2011

Across all studies, the median number of genes tested with ≥3 replicates was 14,900 and the median number of replicates ≥3 was 4 (Fig. 1). Using optimal α instead of α = 0.05 resulted in a reduced probability of the combination of Type I and II errors of 19–29% (Table 1). One important conclusion is that under current practices, tests intended to detect effect sizes of 1 SD will make errors in 5% of tests if there are no treatment effects on any of the genes but the median level of replication (3 replicates per treatment) will make errors in more than 77% of tests if all the genes are affected by the treatment(s) and will make errors in more than 41% of the tests if half the genes are affected by treatment(s). That is, they will maintain the probability of making Type I errors at 0.05 but have highly inflated Type II error probabilities (i.e. low power). For tests intended to detect a 2 SD effect size, again the overall error rate will be 5% if none of the genes are affected by treatment(s) but will be more than 34% with median replication if all the genes are affected by the treatment(s) and almost 20% if half the genes are affected by treatment(s). So, current experimental design practices for microarrays are inadequate, especially with respect to Type II errors, and post-hoc corrections are not mitigating this problem (see below). It is important to note that we do not know the true error rates – for that we would have to know how many and which genes were actually differentially expressed. These are estimated error rates under the assumptions that (1) prior probabilities for H_A_ and H_0_ are equal and (2) critical effect sizes are SD =1,2, or 4.

**Table 1:** Type I and II error rates**:** *Median, 1st and 3rd quartiles, minimum and maximum α, β, average of α and β, and implied costs of Type I/II errors, evaluated for the standard α = 0.05 and for the optimal α approach, at 3 critical effect sizes (1, 2, and 4 SD), for 203 fish microarray papers with tests that have at least 3 replicates, published between 2002 and 2011 (assuming two-tailed, two-sample t-tests)*

Critical effect size	Decision threshold	Statistical parameter	Minimum	1st quartile	Median	3rd quartile	Maximum
1 standard deviation	standard α	α	0.05	0.05	0.05	0.05	0.05
		β	0.088	0.71	0.78	0.84	0.84
		(α + β)/2	0.069	0.38	0.41	0.45	0.45
		Implied Type I/II error cost ratio	1.5	2.6	3.1	3.4	3.9
	optimal α	α	0.064	0.26	0.29	0.32	0.32
		β	0.070	0.34	0.38	0.42	0.42
		(α + β)/2	0.067	0.30	0.33	0.37	0.37
		Implied Type I/II error cost ratio	1	1	1	1	1
2 standard deviations	standard α	α	0.05	0.05	0.05	0.05	0.05
		β	0.0000015	0.21	0.34	0.54	0.54
		(α + β)/2	0.025	0.13	0.20	0.29	0.29
		Implied Type I/II error cost ratio	0.00011	3.4	4.5	5.1	5.1
	optimal α	α	0.0011	0.11	0.15	0.21	0.21
		β	0.00094	0.10	0.13	0.18	0.18
		(α + β)/2	0.0010	0.10	0.14	0.19	0.19
		Implied Type I/II error cost ratio	1	1	1	1	1
4 standard deviations	standard α	α	0.05	0.05	0.05	0.05	0.05
		β	0	0.00023	0.0038	0.052	0.052
		(α + β)/2	0.025	0.025	0.027	0.051	0.051
		Implied Type I/II error cost ratio	0.00011	0.014	0.19	1.9	1.9
	optimal α	α	0.000000031	0.013	0.028	0.065	0.065
		β	0.000000017	0.0065	0.014	0.031	0.031
		(α + β)/2	0.000000024	0.0096	0.021	0.048	0.048
		Implied Type I/II error cost ratio	1	1	1	1	1

### Sample size estimates (within-study replication)

Many microarray and RNA-seq studies (*n* = 3 per treatment) are only appropriate for detecting effects sizes at least of 4 SD at Type I and II error rates of 0.05 or greater. Traditionally, the least conservative acceptable error rates have been set at 0.05 for Type I errors and, when they consider Type II errors, at 0.20 for Type II errors. This implies an average error rate of 0.125 (i.e. [α + β] / 2 ≤ 0.125, the average of α and β associated with using α = 0.05 and achieving 80% statistical power). To detect an effect of 2 SD at an error rate of 0.125 would require sample sizes greater than 5 per treatment, and detecting an effect of 1 SD would require at least 16 samples per treatment (Table 2).

**Table 2:** Replicate estimates: *Number of replicates per treatment needed to achieve maximum acceptable averages of α and β of 0.00001, 0.0001, 0.001, 0.01, 0.05, 0.1, and 0.125, at critical effects sizes of 1, 2, and 4 SD, for an independent two-tailed, two sample t-test*

Maximum acceptable average of α and β	Number of samples required		
	CES = 1SD	CES = 2SD	CES = 4SD
0.00001	156	43	15
0.0001	120	33	12
0.001	85	24	9
0.01	50	14	5
0.05	27	8	3
0.1	18	6	3
0.125	16	5	3

### Repeating the experiment (among- study replication)

Using the optimal α minimize the combined probabilities of Type I and II errors, to reduce the probability of making a Type I error for any particular gene to 0.10 for an effect size of 1 SD using a sample size at the high end of what is usually used in microarray studies (i.e. 10 replicates per treatment), an experiment would have to show a statistically significant effect for a gene in two consecutive experiments. To reduce the probability to 0.001, the experiment would have to show a statistically significant effect for a gene in 4 consecutive experiments. Similarly, to reduce the probability of missing a real effect to 0.10 for an effect size of 1 SD, an experiment with 10 replicates per treatment would have to show no statistically significant effect for a gene in two consecutive experiments. To reduce this probability to 0.001, there would have to be no statistically significant results in 5 consecutive experiments. On the other hand, if the critical effect size is 4 SD, one experiment is all that would be needed for most traditional sample sizes and error rates (Tables 3a and b)

**Table 3:** A and B. Required number of replicates: *A) Number of times a study would have to be repeated with the same conclusion to achieve an α of 0.00001, 0.0001, 0.001, 0.01, 0.05, 0.1, and 0.2, at critical effects sizes of 1, 2, and 4 SD, for an independent two-tailed, two sample t-test. (B) Number of times a study would have to be repeated with the same conclusion to achieve a β of 0.00001, 0.0001, 0.001, 0.01, 0.05, 0.1, and 0.2, at critical effects sizes of 1, 2, and 4 SD, for an independent two-tailed, two sample t-test*

A.								
Critical effect size	Within-study replication	Replication of the experiment needed to achieve						
		α = 0.00001	α = 0.0001	α = 0.001	α = 0.01	α = 0.05	α = 0.1	α = 0.2
1 SD	4	10	8	6	4	3	2	2
	6	9	7	5	4	3	2	2
	8	8	6	5	3	3	2	2
	10	7	6	4	3	2	2	1
2 SD	4	7	5	4	3	2	2	1
	6	5	4	3	2	2	1	1
	8	4	4	3	2	1	1	1
	10	4	3	2	2	1	1	1
4 SD	4	4	3	2	2	1	1	1
	6	3	2	2	1	1	1	1
	8	2	2	2	1	1	1	1
	10	2	2	1	1	1	1	1
B.								
Critical effect size	Within-study replication	Replication of the experiment needed to achieve						
		β = 0.00001	β = 0.0001	β = 0.001	β = 0.01	β = 0.05	β = 0.1	β = 0.2
1 SD	4	12	10	8	5	4	3	2
	6	10	8	6	4	3	2	2
	8	9	7	6	4	3	2	2
	10	8	6	5	3	2	2	2
2 SD	4	6	5	4	3	2	2	1
	6	5	4	3	2	2	1	1
	8	4	3	3	2	1	1	1
	10	4	3	2	2	1	1	1
4 SD	4	3	3	2	2	1	1	1
	6	2	2	2	1	1	1	1
	8	2	2	1	1	1	1	1
	10	2	2	1	1	1	1	1

### Optimal α versus no post-hoc and traditional post-hoc analyses

We used three sets of simulated scenarios of 15,000 tests with 4 replicates per group. The scenarios differed in the assumed prior probability of the null and alternate hypotheses with Scenario 1 assuming a 50% probability of the alternate being true, Scenario 2 a 25% probability and Scenario 3 a 10% probability. Each scenario examined 3 different critical effect sizes, 1, 2, and 4 SD. Optimal α consistently resulted in fewer or the same overall errors when compared to any of the following approaches; no post-hoc test, Bonferroni correction, or an FDR (Table 4A-C).

**Table 4:** A-C. A comparison of the mean number of significant results among four different procedures for evaluating significance of multiple comparisons: *Type I errors, and Type II errors for 100 iterations of 15,000 simulated differential gene expression test using (1) α = 0.05 for all tests, (2) a Bonferroni correction to adjust the family-wise error rate (FWER) to 0.05, (3) the Benjamini-Hochberg procedure to adjust the false-discovery rate (FDR) to 0.05, and (4) optimal α*

Critical effect size (CES)	Average of 100 iterations of 15,000 tests	α = 0.05	Bonferroni FWER = 0.05	Benjamini-Hochberg FDR = 0.05	Optimal α
A.					
CES = 1SD	# of significant results	2046	0	1	6776
	# of Type I errors	376	0	0	2143
	# of Type II errors ≥ CES	5829	7500	7499	2867
	# of Type I and II errors	6205	7500	7499	5010
	% error reduction by using optimal α	19.3%	33%	33%	-
CES = 2SD	# of significant results	5298	3	1709	7659
	# of Type I errors	379	0	43	1130
	# of Type II errors ≥ CES	2581	7497	5834	970
	# of Type I and II errors	2960	7497	5876	2100
	% error reduction by using optimal α	29%	72%	64%	-
CES = 4SD	# of significant results	7848	61	7560	7608
	# of Type I errors	378	0	190	212
	# of Type II errors ≥ CES	30	7439	130	105
	# of Type I and II errors	408	7439	320	317
	% error reduction by using optimal α	22%	96%	1%	-
B.					
CES = 1SD	# of significant results	1400	0	0	1456
	# of Type I errors	562	0	0	590
	# of Type II errors ≥ CES	2912	3750	3750	2883
	# of Type I and II errors	3474	3750	3750	3473
	% error reduction by using optimal α	0.02%	7%	7%	-
CES = 2SD	# of significant results	3032	1	119	3537
	# of Type I errors	562	0	5	791
	# of Type II errors ≥ CES	1280	3749	3636	1004
	# of Type I and II errors	1842	3749	3641	1795
	% error reduction by using optimal α	3%	52%	51%	-
CES = 4SD	# of significant results	4295	31	3665	3826
	# of Type I errors	560	0	136	200
	# of Type II errors ≥ CES	15	3719	221	124
	# of Type I and II errors	575	3719	358	324
	% error reduction by using optimal α	44%	91%	9%	-
C.					
CES = 1SD	# of significant results	1012	0	0	10
	# of Type I errors	680	0	0	5
	# of Type II errors ≥ CES	1167	1500	1500	1495
	# of Type I and II errors	1847	1500	1500	1500
	% error reduction by using optimal α	19%	0%	0%	-
CES = 2SD	# of significant results	1662	1	3	1083
	# of Type I errors	677	0	0	334
	# of Type II errors ≥ CES	515	1499	1497	752
	# of Type I and II errors	1192	1499	1498	1086
	% error reduction by using optimal α	9%	28%	27%	-
CES = 4SD	# of significant results	2169	12	1261	1539
	# of Type I errors	675	0	56	143
	# of Type II errors ≥ CES	6	1488	295	105
	# of Type I and II errors	681	1488	350	248
	% error reduction by using optimal α	64%	83%	29%	-

Type II error rates and optimal α levels were evaluated using three different critical effect sizes (CES), representing effects as large as 1, 2, and 4 standard deviations (SD) of the data. The 15,000 simulated tests had 4 replicates in the experimental and control groups, and were constructed such that (A) H _*A*_ *prior probability = 0.50, H*_*o*_ *prior probability = 0.50; (B) H*_*A*_ *prior probability = 0.25, H*_*o*_ *prior probability = 0.75; (C) H*_*A*_ *prior probability = 0.10, H*_*o*_ *prior probability = 0.90*

Optimal α reduced the number of overall errors (α and β) relative to other approaches by as much as 96%. When the assumed prior probability of H_A_ is low (i.e. 10%) and the critical effect size is small (i.e. 1 SD) Bonferroni and FDR adjustments do as well as optimal alpha because the threshold is so stringent that they find no significant results. Thus, the only error that is made is a  Type II error and these approaches miss all 1500 true effects. Optimal alpha makes slightly fewer Type II error, at 1495. In addition, half of the 10 significant results found using the optimal alpha threshold are false positives resulting in the same number of errors which was 1500 and the same number as for Bonferroni or FDR adjustments. No post-hoc adjustments under these circumstances result in many more true effects being detected but also many more type I errors – more than half of the statistically significant results are false positives. The most conservative post-hoc adjustment, Bonferroni, routinely resulted in the largest overall error rate when the critical effect size was large while not using a post-hoc analysis resulted in fewer errors than either a Bonferroni or FDR except when the prior probability and critical effect size were small. Of course, the distribution of Type I and II errors varies among approaches, with no post hoc adjustment and the FDR adjustment resulting in a relatively large number of Type II errors when the critical effect size was 1 or 2 SD. However, no post-hoc adjustment produced relatively large number of Type I errors when the critical effect size was 4 SD while the FDR approach still resulted in more type II errors. Bonferroni resulted in zero Type I errors but a large number of Type II errors at all effect sizes. Optimal alpha resulted in a much more even distribution of Type I and II errors except when the prior probability and critical effect size was small.

## Discussion

Researchers using high throughput expression techniques enjoy the benefits of global analyses, but must acknowledge the statistical issues associated with an extremely large number of comparisons. Problems may become exacerbated as even higher throughput techniques such as RNA-Seq become more common and genome projects continue to increase the capacity of microarray platforms. RNA-seq experiments are currently restricted due to cost to small sample sizes for each comparison which further exacerbates the error rates. Researchers have generally dealt with the issue of multiple comparisons by using one or more post-hoc adjustments designed to control Type I error rates [18, 19] and it is unlikely that one can publish transcriptomic datasets without using some form of post-hoc correction (e.g. FDR [20], Bonferroni, Tukey’s range test, Fisher’s least significant difference and Bayesian algorithms). Techniques are more conservative (i.e. less likely to result in a Type I error) or less conservative (more likely to result in a Type I error) and implicit in choosing one technique over another is a concern about making a Type II error. That is, the only reason to use a less conservative post-hoc adjustment is if one is concerned about the increasing Type II error rate associated with lowering the probability of making a Type I error. This has, inevitably, led to a large-scale debate that has been relatively unproductive because it is rarely focused on the fundamental issue, that all post-hoc adjustments are designed to reduce Type I error rates (i.e. concluding gene expression has been affected by a treatment when it has not) with little or no explicit regard for the inevitable increase in Type II error rates (i.e. concluding that the treatment has had no effect on gene expression when it has) [21]. Any informed decision about post-hoc adjustments requires a quantitative understanding of both α and β probabilities [22, 23] and a clear assessment of the relative costs of Type I and II errors. However, no post-hoc test currently attempts to explicitly and quantitatively integrate control of Type I and II errors simultaneously and the result is that none of them minimize either the overall error rates or costs of making an error.

One proposed solution to balancing concerns is to set Type I and II error thresholds to be equal [24]. However, the threshold that minimizes the probability of making an error may not be where the Type I and II error probabilities are equal and if Type I and II errors have equal costs, then we should seek to minimize their average probability with no concern for whether the individual probabilities are equal. This is a critical and underemphasized problem in bioinformatics. Our results demonstrate that using optimal α results in reduced error rates compared to using *p* = 0.05 with or without post-hoc corrections. However, it is unlikely that the improvement in error rates attributed to using optimal α will be the same as those estimated here. These results were calculated based on the assumption that the prior probabilities of the alternate being true were 0.5, 0.25 and 0.10, that the costs of Type I and II errors are equal, that the targeted critical effect sizes are 1, 2, or 4 SD and that the results in these 203 studies are representative of all disciplines. But optimal α can accommodate different assumptions about prior probabilities, relative error costs and critical effect sizes and, though the degree to which optimal alpha is superior to traditional approaches may vary, the fundamental conclusion that optimal α error probabilities are as good or better than traditional approaches holds under different assumptions about prior probabilities or critical effect sizes. That said, this is only certain to hold true when we make the assumption implied in null hypothesis testing, that there is either no effect (H_0_) or there is an effect as large as the critical effect size (H_A_).

### Multiple comparisons problem

One particular advantage of optimal α is that it makes post-hoc corrections unnecessary and, in fact, undesirable (correction implies that something desirable has occurred when that isn’t necessarily so – we were tempted to call it a post-hoc distortion). This should dispel some of the complexity and confusion surrounding the analysis of transcriptomic data. However, while optimal α can minimize the errors associated with the large number of comparisons made using microarrays for example, neither optimal α nor any form of post-hoc correction can eliminate the problems associated with multiple comparisons. Any post-hoc test that is done to lower the probability of a Type I error will increase the probability of making a Type II error. While optimal α minimizes the probability of making an error, there will still be an enormous number of unavoidable errors made simply because we are doing a large number of comparisons. There is no simple solution to solving the effects of multiple comparisons of error rates. However, progress can be made by developing new standards for the experimental design of microarray data including large increases in within-study replication, increased among study replication and/or use of ‘network’ approaches which broaden hypotheses to include suites of genes and reduce the total number of hypotheses being tested.

### Experimental design solutions

Our results suggest that standard within-study replication (i.e. 3–8 replicates per treatment) is adequate for critical effect sizes of 2 or 4 SD’s at a target overall error rate of 0.05. Thus, increased replication would only be warranted if detecting smaller effect sizes were desirable. However, we question whether error rates = 0.05 are appropriate when 44 thousand comparisons are being made because a threshold error rate of 0.05 still results in thousands of errors. This is a particular problem when even a handful of statistically significant results might be considered reason enough for publication. We suggest that where thousands of comparisons are being made, the standard for statistical significance must be higher, say, 0.0001 or 0.00001 but not through the use of post-hoc corrections that will increase the probability of Type II errors. To meet these standards and retain high statistical power, within-study replication would require dramatic increases in the number of biological replicates used in experiments. Currently, the standard appears to be 3–8 biological replicates per treatment. Replication is limited by a variety of factors including financial costs, available person hours, sample availability, and physical space. However, where possible, it would often be preferable to test fewer genes using much larger samples sizes, especially because the price of microarrays/NGS will likely to continue to drop, making replication of 50, 100 or 200 possible and, in some cases, warranted. These may seem like drastic replication recommendations but the problems associated with high throughput of molecular data are unusual and perhaps unprecedented and it is not surprising that when the number of comparisons that can be made at one time is large the number of replicates per comparison will also need to be large. Our results suggest that the number of replicates per treatment required to maintain acceptable error rates is large, and estimated to be 15–150, depending on the critical effect size.

An alternative approach is to replicate experiments rather than increasing the number of replicates within an experiment. This would involve identifying the number of times one would have to see the same result repeated, given a particular experimental design, before we would be willing to accept the result. Our results suggest that if 8–10 replicates per treatment are used and the critical effect size is 2 or 4 SD’s then an experiment would not need to be repeated to meet at error rate = 0.05. However, the argument for a more stringent acceptable error rate applies here as well and so at an error rate of 0.0001 experiments would need to be repeated 2–10 times. It’s not clear whether increased within-experiment or among experiment replication would be more efficient and may depend on the limiting factors in particular labs. However, it is clear that among-experiment replication adds an additional layer of inferential complexity because it would require interpretation of cases where a subset, but not all, of the experiments was consistent.

It’s not clear whether within- or among-study replication is preferable – which is preferable may depend on context – but it is clear that one or both are necessary if conclusions of microarray studies are to be rigorous and reliable.

### Relative costs of type I and II errors

All of our analyses assumed that the costs of Type I and II errors were equal but we do not preclude the possibility that Type I and II errors should be weighted differently [25–27] and an additional advantage of optimal α is that the cost of error can be minimised rather than the probability of error. Thus, if the relative costs of Type I and II error can be estimated they can be integrated into the selection of appropriate statistical thresholds. The question of relative costs of Type I and II errors is a difficult and relatively unexplored one but the objectives of a study can often guide setting relative costs of Type I and II error. For example, preliminary work ‘fishing’ for genes that may respond to a specific treatment might be more concerned about missing genes that were actually affected than identifying genes as affected when they really weren’t and would choose to set the cost of Type II errors greater than Type I errors. By contrast, a researcher attempting to identify a single gene (i.e. biomarker) that is regulated by a specific treatment or drug might decide that Type I error is a larger concern.

Post-hoc multiple comparison adjustments to reduce Type I errors at the expense of increased Type II errors imply that Type I errors are more serious than Type II errors. Although we don’t believe Type I errors are actually more serious than Type II errors under all circumstances in which multiple comparison α adjustments have been used, similar outcomes can be obtained through the optimal α approach by selecting a large Type I / Type II error cost ratio a priori.

### SD versus k-fold effect size

One potential complication is that the convention in microarray analysis is often to use effect sizes expressed as multiples of the control mean (e.g. 1-fold, 2-fold or 4-fold change). Unlike effect sizes measured in standard deviations, which combine the raw effect size with the variability of the data, k-fold effect sizes, even when combined with a statistical significance threshold, do not quantitatively incorporate variability in the data. This implies that if a constant k-fold effect size was set as the critical effect size, say 2-fold, that there would be a different optimal α for each comparison, depending on the variability in expression of each gene. That is, if there were 44,000 comparisons, it would require 44,000 different optimal α levels. However, if k-fold effect sizes are used, solutions include (i) setting a single optimal α based on the ‘average’ variability across genes, (ii) grouping genes into variability categories and using a single optimal α for each category based on the average variability in each category, and (iii) setting an individual optimal α for each gene. While both –k-fold and SD effect sizes are reasonable indices of effects, using SD simplifies the application of optimal alpha. However, for microarray and RNA-seq, there are approaches such as Voom [28] that would allow using both k-fold changes and a single optimal alpha for all genes by making the variance homoscedastic.

### A priori H_0_ and H_A_ probabilities

The accuracy of estimates of optimal alpha is a function of assumptions about the a priori probabilities of H_0_ and H_A_. It is inarguably true that estimates of optimal alpha will be less reliable if estimates of a priori H_0_ and H_A_ probabilities are inaccurate. Thus, research into prior probabilities of global gene expression is critical. One key advantage of optimal alpha is that it has the explicit objective of identifying the threshold that will result in the lowest probability of cost of making a mistake. There is, indisputably, a true optimal alpha – if we know the true a priori probabilities of H_0_ and H_A_ and the true relative costs of Type I and II errors then the statistical threshold that minimizes the probability or cost of making an error can be calculated for any target critical effect size. By contrast, the approach of alpha = 0.05 with post hoc corrections (i.e. Bonferroni or FDR) doesn’t explicitly address Type II errors, it only explicitly addresses Type I errors. Of course, there is an implicit concern about Type II error – if there wasn’t we would simply always set alpha = 0 and never make a Type I error. We don’t set alpha = 0.00 because the goal is to make as few type I errors as possible *while* also detecting true effects. This implies that we should explicitly address (1) Type II errors, (2) the balance between Type I and II errors and (3) the assumptions about a priori H_0_ and H_A_ probabilities and relative costs of Type I and II errors. Optimal alpha does this and alpha = 0.05 with or without post-hoc corrections does not.

### Application

We have included R code (Additional file 2: Appendix S1) that can be used to calculate optimal α for t-tests ANOVA’s and regressions. To apply optimal α to microarray analysis, you must choose the type of test, sample size, critical effect size, a priori null/alternate probabilities and relative costs of Type I/II errors.

1. Type of test: We have written code for t-tests, ANOVA’s and regressions. Here we have focused on t-tests. The code allows the choice of 1 or 2-tailed tests and one-, two- or paired-sample tests.
2. Sample size: This is usually limited by time and/or money. Most microarray studies test many genes with relatively few replicates (3–6) and the result is a large number of false positives and negatives due to insufficient power. A different experimental design may be desired, testing fewer genes with a higher number of replicates (12–24) to reduce the number of errors. This includes a priori hypotheses for specific pathways perturbed by a stressor.
3. Critical effect size type: This can be measured in standard deviations (e.g. 1, 2, and 4 SD’s), absolute differences (e.g. difference in signal intensity) or relative differences (e.g. fold differences between treatment and control samples – 0.5, 1, 2× more/less gene expression). SD’s are less labour intensive than absolute r relative differences because you set a single optimal α for all genes. If you target absolute or relative critical effects, it will require a separate optimal α for each gene tested in the microarray. This is because the variability among replicates for different genes will vary and therefore the power to detect the same absolute or fold difference will vary among genes.
4. Critical effect size value: A single critical effect size or a range of critical effect sizes can be chosen. Unless there is a defensible reason for choosing a single critical effect size, we suggest selecting a range of critical effect sizes that span small, moderate and large effects. Each critical effect will have a different optimal α (i.e. optimal α for large effects will be smaller than for moderate effects which will be smaller than for small effects) and this allows for different conclusions about small versus moderate versus large effects. For example, it would be reasonable to conclude that there is evidence for small effects but no evidence for moderate or large effects.
5. A priori probability of null and alternate hypotheses: There must be explicit quantitative probabilities for these values. We suggest assuming these to be equal (i.e. 0.5 for both) unless you have theoretical or empirical reasons to believe otherwise.
6. Relative costs of Type I and II errors: It is not unreasonable to expect that the costs of missing a real effect could be different than detecting a false effect and such difference can be assigned in the code for calculating optimal α. However, unless there is a clear, explicit reason for estimating the costs to be different, we recommend assuming equal costs of Type I and II errors.

## Conclusions

While we don’t have empirical estimates of the true error rates associated with the studies used in the meta-analysis, both the meta-analysis and simulations estimated error rates under simple and reasonable assumptions and suggest that optimal α provides a simple and superior approach to setting statistical thresholds for transcriptome analysis than the traditional α = 0.05 with or without post-hoc adjustments. Using optimal α will provide significantly lower probabilities of making errors and will eliminate the need to use complex and controversial post-hoc adjustments. However, optimal α cannot eliminate the problems associated with the large number of tests that are traditionally carried out in transcriptome analysis. This problem will only become exacerbated as new high-throughput techniques such as RNA-Seq become more commonly used and increasing amounts of information are generated. Thus, moving forward, researchers should consider setting new standards for within and among-study replication and exploring novel approaches to evaluating gene expression data such as in the case of gene enrichment analyses.

## Additional files

Additional file 1: Data S1. Description of studies used in the meta-analysis. (XLSX 45 kb)

Additional file 2: Appendix S1. R-code for optimal alpha estimates for t-tests, ANOVA’s, and regressions. (DOCX 15 kb)

Additional file 3: Data S2. Raw and summarized simulation data. (XLSX 8649 kb)

## Abbreviations

ANOVA: Analysis of Variance
DEG: Differentially Expressed Genes
FDR: False Discovery Rate
NGS: Next Generation Sequencing
RNA-seq: RNA sequencing
SD: Standard Deviation
