# Supplementary material for: Optimal alpha reduces error rates in gene expression studies: a meta-analysis approach
Source: BMC Bioinformatics. 2017 Jun 21;18:312. doi: 10.1186/s12859-017-1728-3 (PMC5480162; doi:10.1186/s12859-017-1728-3)
Supplement: Supplementary file 2 — Appendix S1. R-code for optimal alpha estimates for t-tests, ANOVA’s, and regressions. (DOCX 15 kb) [file 12859_2017_1728_MOESM2_ESM.docx]

Appendix 1

R code for calculating optimal alpha for t-tests:

#Optimal alpha t-test R code version 1.1, updated for compatibility with R version 3.x.

#Authored by Joe Mudge (questions or comments? contact: joe.mudge83@gmail.com).

optab<-function (n1=NULL,n2=NULL,d=NULL,T1T2cratio=1,HaHopratio=1,type = c("two.sample", "one.sample", "paired"),tails = c("two.tailed","one.tailed")) {

beta.t.test<-function (n1 = NULL, n2 = NULL, d = NULL, sig.level = 0.05, type = c("two.sample", "one.sample", "paired"),tails = c("two.tailed","one.tailed")) {

if (!is.null(sig.level) && !is.numeric(sig.level) || any(0 > sig.level | sig.level > 1))

stop(sQuote("sig.level"), " must be numeric in [0, 1]")

if (!is.null(n1) && n1 < 2)

stop("number of observations in the first group must be at least 2")

type <- match.arg(type)

tails <- match.arg(tails)

d<-abs(d)

tsample <- switch(type, one.sample = 1, two.sample = 2, paired = 1)

tside <- switch(tails, one.tailed = 1, two.tailed = 2)

if (tside == 1) {

p.body <- quote({

nu <- switch(type, one.sample = n1-1, two.sample = n1 + n2 - 2, paired = n1-1)

pt(qt(sig.level/tside, nu, lower = FALSE), nu, ncp = d * switch(type, one.sample = sqrt(n1), two.sample = (1/sqrt(1/n1 + 1/n2)), paired = sqrt(n1)), lower = FALSE)

})

}

if (tside == 2) {

p.body <- quote({

nu <- switch(type, one.sample = n1-1, two.sample = n1 + n2 - 2, paired = n1-1)

qu <- qt(sig.level/tside, nu, lower = FALSE)

pt(qu, nu, ncp = d * switch(type, one.sample = sqrt(n1), two.sample = (1/sqrt(1/n1 + 1/n2)), paired = sqrt(n1)), lower = FALSE) +

pt(-qu, nu, ncp = d * switch(type, one.sample = sqrt(n1), two.sample = (1/sqrt(1/n1 + 1/n2)), paired = sqrt(n1)), lower = TRUE)

})

}

1-eval(p.body)

}

w.average.error<-function (alpha=NULL,n1=NULL,n2=NULL,d=NULL,T1T2cratio=1,HaHopratio=1,type = c("two.sample", "one.sample", "paired"),tails = c("two.tailed","one.tailed"))

((alpha*T1T2cratio+HaHopratio*(beta.t.test(n1=n1,n2=n2,d=d,sig.level=alpha,type=type,tails=tails))))/(HaHopratio+T1T2cratio)

min.average.error<-function (n1=NULL,n2=NULL,d=NULL,T1T2cratio=1,HaHopratio=1,type = c("two.sample", "one.sample", "paired"),tails = c("two.tailed","one.tailed"))

unlist(optimize(w.average.error,c(0,1),tol=0.0000000000001,n1=n1,n2=n2,d=d,T1T2cratio=T1T2cratio,HaHopratio=HaHopratio,type=type,tails=tails))[2]

alpha<-function (n1=NULL,n2=NULL,d=NULL,T1T2cratio=1,HaHopratio=1,type = c("two.sample", "one.sample", "paired"),tails = c("two.tailed","one.tailed"))

unlist(optimize(w.average.error,c(0,1),tol=0.000000000001,n1=n1,n2=n2,d=d,T1T2cratio=T1T2cratio,HaHopratio=HaHopratio,type=type,tails=tails))[1]

beta<-function (n1=NULL,n2=NULL,d=NULL,T1T2cratio=1,HaHopratio=1,type = c("two.sample", "one.sample", "paired"),tails = c("two.tailed","one.tailed"))

((T1T2cratio+HaHopratio)*min.average.error(n1=n1,n2=n2,d=d,T1T2cratio=T1T2cratio,HaHopratio=HaHopratio,type=type,tails=tails)-T1T2cratio*alpha(n1=n1,n2=n2,d=d,T1T2cratio=T1T2cratio,HaHopratio=HaHopratio,type=type,tails=tails))/HaHopratio

list(

"test type"=match.arg(type),

"tails"=match.arg(tails),

"output"=t(data.frame(

"sample size 1"=n1,

"sample size 2"=n2,

"Cohen's d effect size"=d,

"Type I/II error cost ratio"=T1T2cratio,

"Ha/Ho prior probability ratio"=HaHopratio,

"overall probability of error"=(alpha(n1=n1,n2=n2,d=d,T1T2cratio=T1T2cratio,HaHopratio=HaHopratio,type=type,tails=tails)+HaHopratio*beta(n1=n1,n2=n2,d=d,T1T2cratio=T1T2cratio,HaHopratio=HaHopratio,type=type,tails=tails))/(1+HaHopratio),

"cost-weighted probability of error"=min.average.error(n1=n1,n2=n2,d=d,T1T2cratio=T1T2cratio,HaHopratio=HaHopratio,type=type,tails=tails),

"optimal alpha"=alpha(n1=n1,n2=n2,d=d,T1T2cratio=T1T2cratio,HaHopratio=HaHopratio,type=type,tails=tails),

"optimal beta"=beta(n1=n1,n2=n2,d=d,T1T2cratio=T1T2cratio,HaHopratio=HaHopratio,type=type,tails=tails),row.names="values"))

)

}

#The function used to calculate optimal alphas is: optab(n1=NULL,n2=NULL,d=NULL,T1T2cratio=1,HaHopratio=1,type = c("two.sample", "one.sample", "paired"),tails = c("two.tailed","one.tailed"))

#The arguments 'n1' and 'n2' are the samples sizes of each group (for a one sample test, enter any value >=3 for n2, n2 will be ignored)

#The argument 'd' is the 'Cohen's d' standardized critical effect size. Cohen's d = difference between group means/pooled within group standard deviation

#The argument 'T1T2cratio' is the cost ratio of Type I errors relative to Type II errors. T1T2cratio is set at 1 as a default, making Type I and Type II errors equally serious.

#The argument 'HaHopratio' is the prior probability of the alternate hypothesis relative to the prior probability of the null hypothesis. HaHopratio is set at 1 as a default, to not weight alpha and beta by their prior probabilities (assuming they are unknown).

#The argument 'type' is the type of t-test being undertaken and must be "two.sample", "one.sample" or "paired". If ignored, "two.sample" is the default.

#The argument 'tails'is the number of tails being examined and must be either "two.tailed" or "one.tailed". If ignored, "two.tailed" is the default.

#This code is partially based on code modified from the R package 'pwr'(Champely 2009).
